# Genome-Wide Association Study of Serum Selenium Concentrations

**DOI:** 10.3390/nu5051706

**Published:** 2013-05-21

**Authors:** Jian Gong, Li Hsu, Tabitha Harrison, Irena B. King, Stefan Stürup, Xiaoling Song, David Duggan, Yan Liu, Carolyn Hutter, Stephen J. Chanock, Charles B. Eaton, James R. Marshall, Ulrike Peters

**Affiliations:** 1 Public Health Sciences Division, Fred Hutchinson Cancer Research Center, Seattle, WA 98109, USA; E-Mails: jgong@fhcrc.org (J.G.); lih@fhcrc.org (L.H.); tharriso@fhcrc.org (T.H.); xsong2@fhcrc.org (X.S.); 2 Department of Biostatistics, University of Washington, Seattle, WA 98195, USA; 3 Department of Internal Medicine, University of New Mexico, Albuquerque, NM 87131, USA; E-Mail: IKing@salud.unm.edu; 4 Department of Pharmacy, University of Copenhagen, Copenhagen, DK-2100, Denmark; E-Mail: stefan.sturup@sund.ku.dk; 5 Translational Genomics Research Institute, Phoenix, AZ 85004, USA; E-Mail: dduggan@tgen.org; 6 Stephens and Associates, Carrollton, TX 75006, USA; E-Mail: yliu@stephens-associates.com; 7 Division of Cancer Control and Population Sciences, National Cancer Institute, Bethesda, MD 20892, USA; E-Mail: huttercm@mail.nih.gov; 8 Division of Cancer Epidemiology and Genetics, National Cancer Institute, Bethesda, MD 20892, USA; E-Mail: hanocks@mail.nih.gov; 9 Center for Primary Care and Prevention, Memorial Hospital of Rhode Island, Pawtucket, RI 02860, USA; E-Mail: Charles_Eaton@mhri.org; 10 Department of Health Behavior, Roswell Park Cancer Institute, Buffalo, NY 14263, USA; E-Mail: James.Marshall@RoswellPark.org; 11 Department of Epidemiology, School of Public Health, University of Washington, Seattle, WA 98195, USA

**Keywords:** selenium, serum, selenoprotein, genome-wide association study, AGA, NEIL3, SLC39A11

## Abstract

Selenium is an essential trace element and circulating selenium concentrations have been associated with a wide range of diseases. Candidate gene studies suggest that circulating selenium concentrations may be impacted by genetic variation; however, no study has comprehensively investigated this hypothesis. Therefore, we conducted a two-stage genome-wide association study to identify genetic variants associated with serum selenium concentrations in 1203 European descents from two cohorts: the Prostate, Lung, Colorectal, and Ovarian (PLCO) Cancer Screening and the Women’s Health Initiative (WHI). We tested association between 2,474,333 single nucleotide polymorphisms (SNPs) and serum selenium concentrations using linear regression models. In the first stage (PLCO) 41 SNPs clustered in 15 regions had *p* < 1 × 10^−5^. None of these 41 SNPs reached the significant threshold (*p* = 0.05/15 regions = 0.003) in the second stage (WHI). Three SNPs had *p* < 0.05 in the second stage (rs1395479 and rs1506807 in 4q34.3/*AGA*-*NEIL3*; and rs891684 in 17q24.3/*SLC39A11*) and had p between 2.62 × 10^−7^ and 4.04 × 10^−7^ in the combined analysis (PLCO + WHI). Additional studies are needed to replicate these findings. Identification of genetic variation that impacts selenium concentrations may contribute to a better understanding of which genes regulate circulating selenium concentrations.

## 1. Introduction

Selenium, an essential trace element, has been suggested to influence various health outcomes, particularly cancer [1,2,3]. Selenium deficiency causes Keshan disease (cardiomyopathy) and Kashin-Beck disease (osteoarthritis) and has been linked to oxidative stress, inflammation, and cancer [1,4,5,6,7,8]. Selenium is needed for normal immune function and is involved in viral suppression [9,10,11]. Adequate selenium intake is important for sperm maturation, thyroid hormone synthesis, and may delay the aging process [12,13,14,15].

Blood selenium concentrations tend to vary substantially [16] and are influenced by both exogenous factors such as diet, supplements, or smoking status, as well as endogenous factors such as selenium storage, transport and excretion [1,17,18]. Identification of genetic variation that impacts selenium concentrations may contribute to better understanding of which genes impact endogenous factors that affect circulating selenium concentrations, ultimately, leading to improved prevention of selenium-related health outcomes. 

Studies showed that genetic variation in GPX1 may change enzyme activity, correlation between GPX1 activity and selenium concentrations and impacts overall selenium concentrations after supplementations. These findings may suggest that genetic variants in selenoproteins impact circulating selenium concentrations. Glutathione peroxidase 1 (GPX1) is not only important for the anti-oxidative properties of selenium in the human body but also impacts selenium storage [19]. Studies showed that genetic variants in the *GPX1* gene can change its enzyme activity [20,21,22], leading to changes in plasma selenium concentrations [23,24]. Selenoprotein P (SEPP1) is estimated to contain at least 40% of total plasma selenium [25] and, hence, has a central role in selenium transport [17]. Genetic variants in *SEPP1* appear to impact functions and synthesis of SEPP1 and affect activity of other selenoproteins (e.g., GPX1) [26,27,28], which might result in changes in selenium concentrations. To date only a limited set of genetic variants have been investigated, and there is no comprehensive evaluation of the impact of genetic variants across the genome on circulating selenium concentrations. In this study, we conducted a two-stage genome-wide association study (GWAS) by using the data from two cohorts to examine the effects of genetic variation on serum selenium concentrations. 

## 2. Subjects and Methods

### 2.1. Study Population

This study is based on two cohorts with measurements of serum selenium concentrations and genome-wide association study data: (1) the Prostate, Lung, Colorectal and Ovarian Cancer Screening Trial (PLCO), a large population based randomized trial designed to determine the effects of screening on cancer-related mortality and secondary endpoints [29], and (2) the Women’s Health Initiative (WHI) observation study, a long-term national health study that has focused on strategies for preventing heart disease, cancer, and osteoporotic fractures in postmenopausal women [30]. In PLCO, participants for this analysis were previously selected for a nested-case-control study for colorectal cancer to conduct a GWAS analysis as well as to measure serum selenium. Five hundred and eighty-two PLCO participants with genotyping data and serum selenium concentrations available were included in this study. Similarly, participants in WHI had availability of both genotyping data and serum selenium measurements as part of a nested case-control study of colorectal cancer (*n* = 621). Both studies were restricted to participants of European descent because the number of participants of non-European descent was too small to allow a stratified analysis. All participants gave informed consent, and studies were approved by the Institutional Review Boards at respective institutes. 

### 2.2. Genotyping and Serum Selenium Measurement

DNA was extracted from blood samples using conventional methods. Genotyping was completed using Illumina HumanHap 550K, 610K, or combined Illumina 300K and 240K BeadChip Array System (Illumina, Inc., San Diego, CA, USA). Details of genotyping process, quality-control assess of genotypes are described previously [31]. In brief, samples with <98% completion were excluded. Genotyped single nucleotide polymorphisms (SNPs) were excluded based on call rate (<98%), lack of Hardy Weinberg Equilibrium in controls (HWE, *p* < 1 × 10^−4^), and low minor allele frequency (MAF ≤ 1%).

As imputation of genotypes is established as standard practice in the analysis of genotype array data, all autosomal SNPs of both studies were imputed to the CEU population (Caucasian residents of European ancestry from Utah, USA) in HapMap II release 24 using MACH (Markov Chain Haplotyping algorithm) [32]. Imputed data were merged with genotype data such that genotype data were preferentially selected if a SNP had both types of data, unless there was a difference in terms of reference allele frequency (>0.1) or position (>100 base pairs), in which case imputed data were used. As a measurement of imputation accuracy we calculated R^2^. Imputed SNPs were restricted based on MAF > 1% and imputation accuracy *R*^2^ > 0.3. In total we included 2,474,333 SNPs (either directly genotyped or imputed) in the genome-wide analysis.

In PLCO, serum selenium concentrations were determined by using an inductively coupled plasma mass spectrometry method [33]. Blinded quality control samples (15%) were randomly inserted within each batch and monitored throughout the analysis [34]. The coefficient of variance (CV), estimated from the blinded duplicates, was 9.4%. In WHI, serum selenium levels were measured using atomic absorption spectrometry (Perkin Elmer, Fremont, CA, USA) [35,36,37]. Blinded quality control samples (6%) were included in batches with study samples. The mean CV for the blinded duplicates was 5.8% [38]. Each sample was run in duplicate and considered acceptable if the CV was less than 10%. Internal quality control samples were run before and after each batch to ensure the quality of essay [38].

### 2.3. Statistical Analysis

We used linear regression models assuming additive effects for genotyped SNP (codes 0, 1, and 2 for the number of variant) and using the expected number of variants for imputed SNPs (a number between 0 and 2) to examine the associations between SNPs and serum selenium concentrations. Serum selenium concentration was transformed by taking a nature log due to its positively skewed distribution. We used a two stage design, where we selected the most significant SNPs (those with *p* < 1 × 10^−5^) from the first stage analysis in PLCO for the second stage analysis in WHI, which allows for an independent validation of findings from the first stage. SNP that were taken into the second stage were defined as statistical significant if they reached a *p*-value < 0.05/15 of regions for the SNPs from the first stage. We adjusted for number of regions rather than SNPs selected from the first stage to account for the correlation between SNPs within each region. As secondary analysis we conducted combined analyses across PLCO and WHI for SNPs selected in the first stage analysis. In the first and second stage, we ran linear regression models with adjustment for age, BMI, smoking status (ever *vs.* never smoker), cancer status based on nested case-control study (Yes/No), and the first three principle components of ancestry to examine the effects of SNP on serum selenium concentrations. In combined analyses, we pooled the two cohorts together while adjusting for the same variables as in the two-stage analysis as well as a cohort indicator variable. LocusZoom plots [39] were used to graphically show the GWAS results within a given genomic region. All analyses were performed using the R software (version 2.14.0). All statistical tests were two-sided.

We generated quantile-quantile (Q-Q) plots to assess whether the distribution of the *p* values was consistent with the null distribution (except for the extreme tail). We also calculated the genomic inflation factor (λ) to measure the over-dispersion of the test statistics from the association tests by dividing the median of the squared *Z* statistics by 0.455, the median of a chi-squared distribution with 1 degree of freedom. The inflation factor λ was 1.09 based on all SNPs including both directly genotyped and imputed, indicating there is little evidence of residual population substructure, cryptic relatedness, or differential genotyping between cases and controls. This result was consistent with the visual inspection of the Q-Q plot (Supplementary Figure S1).

## 3. Results

The study characteristics of 582 PLCO and 621 WHI participants are shown in Table 1. As both cohorts focused on chronic disease participants tend to be older (the average age is 64 years for PLCO and 67 years for WHI). Also the BMI was very similar across cohorts, while the fraction of smokers was slightly higher in PLCO than in WHI as can be expected given that we only included men from the PLCO study which tend to smoke more than women. Average serum selenium concentrations were similar and relatively high in both cohorts.

**Table 1:** The characteristics and serum selenium levels in PLCO and Women’s Health Initiative (WHI).

Variable	PLCO ( *n* = 582 men)	WHI ( *n* = 621 women)	PLCO + WHI ( *n* = 1203)
Age (years)	64 (5) ^a^	67 (7)	65 (6)
BMI (kg/m^2^)	27.5 (3.9)	27.8 (5.5)	27.7 (4.8)
Ever smoker (Yes/No)	363 (62) ^b^	343 (55)	706 (59)
Cancer status (Yes/No)	20 (3)	584 (94)	604 (50)
Selenium levels (mg/dL)	142.4 (27.0)	138.1 (21.3)	140.2 (24.3)

^a^ mean (SD); ^b^ *n* of Yes (%).

**Figure 1:**
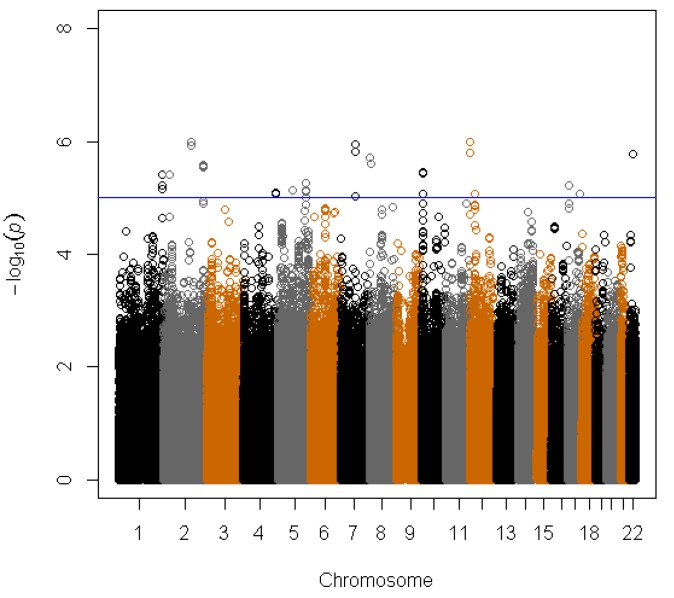
Manhattan plot of the genome-wide scan in Prostate, Lung, Colorectal, and Ovarian Cancer Screening Trial (PLCO) (*n* = 582). The −log10 of *p* values for 2,474,333 single nucleotide polymorphisms (SNPs) plotted against physical chromosomal positions. SNPs above the blue line represent those with a *p* value <1 × 10^−5^.

**Figure 2:**
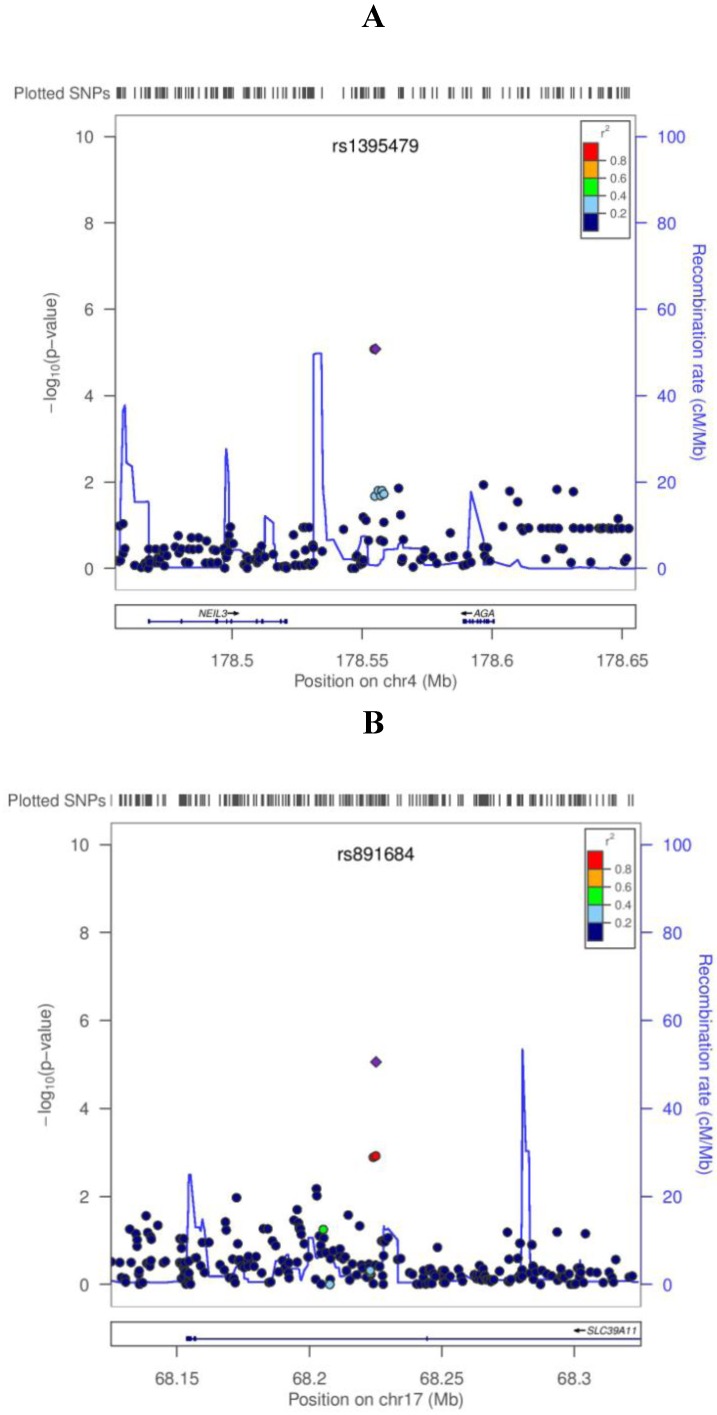
LocusZoom plots for the two loci associated with serum selenium concentrations at *p* value < 5 × 10^−7^ in the combined analysis (**A**: 4q34.3; **B**: 17q24.3). In each plot, the −log10 of *p* values are on the left *y*-axis; the SNP genomic position (NCBI build 36) on the *x*-axis; the estimated recombination rate from HapMap II CEU are on the right *y*-axis and plotted in blue. The most significant SNP was denoted with purple diamond and plotted using the *p* value attained from the genome-wide scan in PLCO. SNPs are colored to reflect linkage disequilibrium (LD) with the most significant SNP (pairwise *r*^2^ from HapMap II CEU). Gene annotations are from the UCSC genome browser.

To display the results of the regression test of all 2.47M tested SNPs with serum selenium concentration with the PLCO cohort we generated a Manhattan plot (Figure 1). Overall, 41 SNPs had *p* values <1 × 10^−5^. These 41 SNPs clustered in 15 regions (1q43, 2p22.3, 2q23.3, 2q35, 4q34.3, 5q14.3, 5q33.2, 7q21.11, 8p23.2, 10p14, 12p13.33, 12p11.23, 17p12, 17q24.3, and 22q12.3) (Supplementary Table S1). Among these, the SNP rs119902616 in 2q23.3 located in the intronic region of *NEB* gene had the smallest *p* value 1.0 × 10^−6^ based on the PLCO only results. All 41 SNPs were tested in the second stage. However, none of the 41 SNPs reached the significance threshold of 0.003 (0.05/15 regions) in the second stage. Three of the 41 SNPs in two regions (rs1395479 and rs1506807 in 4q34.3 and rs891684 in 17q24.3) had *p* < 0.05 in the second stage with combined *p* < 5 × 10^−7^ in the joint analysis of PLCO and WHI (Table 2; Supplementary Table S1). For all three SNPs with combined *p* values <5 × 10^−7^ was the beta-estimates for serum selenium in the same direction for both cohorts with slightly weaker effects in WHI compared with PLCO (Table 2). To show associations of surrounding SNPs in both regions (4q34.3 and 17q24.3) we used LocusZoom plots as shown Figure 2a,b. In the 4q34.3 region, rs1395479 and rs1506807 are highly correlated with each other (*r*^2^ = 1.0) and show similar low *p* values for the association with selenium concentrations. In the 17q24.3 region, rs891684 has the smallest p value. This SNP was in high LD (*r*^2^ > 0.8) with 3 SNPs (rs9899648, rs2567504, and rs16977351) that also showed low *p* values for an association with selenium concentrations.

**Table 2:** Three SNPs associated with serum selenium concentrations at *p* values <5 × 10^−7^ in combined analysis.

SNP	Position ^a^	Gene ^b^	Coded allele	CAF ^c^	PLCO		WHI		Combined analysis	
					beta	*p* value	beta	*p* value	Beta	*p* value
rs1506807	4:178554686	*AGA*	A	0.73	−0.059	8.39E−06	−0.025	1.32E−0	−0.043	2.63E−07
rs1395479	4:178555185	*AGA*	A	0.27	0.059	8.31E−06	0.025	1.33E−02	0.043	2.62E−07
rs891684	17:68225134	*SLC39A11*	A	0.05	−0.133	8.73E−06	−0.057	9.99E−03	−0.093	4.04E−07

^a^ NCBI build 36 (chromosome: base pair position); ^b^ gene or near gene; ^c^ coded allele frequency.

Due to a prior hypothesis that some selenoproteins may impact selenium concentration we also examined the SNPs within 200 kb up or downstream of *GPX1* and *SEEPP1* genes using the GWAS data in PLCO and WHI. In the combined analysis of PLCO and WHI, 44 SNPs surrounding *GPX1* (1 kb–180 kb up/downstream) were associated with selenium concentrations at *p* values ranging from 0.01 to 0.05. Furthermore, we observed 39 SNPs within or 0.5–190 kb up/downstream of *SEPP1* to be correlated with selenium concentrations with p values ranging from 0.02 to 0.05. All SNPs with *p* value <0.05 in combined analyses showed effect estimates in the same direction across both cohorts.

## 4. Discussion

In this study, we used a two-stage design to take advantage of the GWAS data from two independent populations to examine the association between genetic variants and serum selenium concentrations. In the first stage we observed 15 regions associated with serum selenium concentrations at *p* < 1 × 10^−5^. However, none of the regions reached the significance threshold in the second stage. Only two regions (4q34.3 and 17q24.3) had *p* < 0.05 in the second stage; and in the joint analysis the associations the two regions has *p* < 5 × 10^−7^ but did not reach the conventional genome-wide significance level of *p* < 5 × 10^−8^. However, as has been previously shown [40], a large fraction of SNPs with borderline genome-wide significant associations replicated when results from additional studies were added, suggesting that further follow-up of these two regions is warranted.

Interestingly the most significant SNP rs1395479 in 4q34.3 was also found to be associated with heart rate traits (*p* = 6.9 × 10^−6^) in a genome-wide scan conducted within the Framingham Heart Study [41]. rs1395479 is located in an intergenic region between the aspartylglucosaminidase (*AGA*) gene (~33 kb downstream) and the *NEIL3* gene (~35 kb downstream). AGA is involved in the lysosomal breakdown of glycoproteins [42]. Glycoproteins occur in the cytosol, cell membrane, and extracellular space [43]. They are equipped with an extremely large number of functions such as transport molecule, function as hormones, enzymes (oxidoreductases, transferases, and hydrolases), and receptors (adhering cells to cells and cells to substratum) [44,45,46,47,48]. Due to the diverse functions of glycoproteins, they appear in nearly every biological process studied [44,45,46,47,48]. It is of interest that SEPP1 that plays a central role in selenium transport is a highly glycosylated protein and AGA might influence selenium concentration through its effects on glycosylation which might change SEPP1’s secretion from endoplasmic reticulum, interaction with chaperons, or catabolism [49]. If this finding is replicated, it may provide evidence that *AGA* impacts serum selenium concentrations through its regulation and control of glycoproteins. NEIL3 belongs to a class of DNA glycosylases and is involved in DNA repair by cleaving damaged bases [50]. Whether it is related to cancer is still unknown. No association between selenium metabolism and NEIL3 has been reported. Thus, it remains unclear at this point if genetic variation in *NEIL3* affects selenium concentrations. 

In 17q24.3, the most significant SNP rs891684 is located in an intron of the gene *SLC39A11*. The function of *SLC39A11* is not well described but it was found to be associated with survival of amyotrophic lateral sclerosis [51] and visceral adipose tissue [52] in previous GWAS. Additionally, the 17q24.3 locus was found to be associated with prostate cancer [53]. Interestingly, animal studies suggested that adipose tissue may be related to selenium storage and selenium levels are associated with obesity [54,55], which may provide further support for an association between genetic variations in *SLC39A11* and circulating selenium concentrations. 

Selenoproteins which incorporate selenium into their active center play in an important role in selenium metabolism in human body. For instance, GPX1 is important for selenium storage [19] and SEPP1 plays a central role in selenium transport [17]. We found that SNPs within or up/downstream of *GPX1* and *SEPP1* were nominally associated with serum selenium concentrations (*p* = 0.01–0.05), and that the directions of effect estimates for these SNPs are consistent between the two cohorts. Considering the usually moderate effects of common genetic variants, power of our study may limit us to detect the associations among SNPs in *GPX1* and *SEPP1*.

Our study has several strengths. To the best of our knowledge, this is the first genome wide scan on circulating selenium concentrations. We used a two-stage design and followed it with a joint analysis in two cohorts to reduce the likelihood of false positive results. The three SNPs with *p* < 5 × 10^−7^ in the joint analysis showed no evidence for heterogeneity in SNP-selenium association between two cohorts. However, our study also has limitations. In PLCO and WHI, average serum selenium concentrations were relative high and there were few participants with selenium-deplete concentrations. If the impact of genetic variation is largest in subjects with low selenium concentrations, we expect that our power to identify selenium-related genetic variants would be improved if we had included more subjects with low serum selenium concentrations. About 94% of the WHI participants were cancer cases and their selenium levels may not represent those from women without cancers, although we did not observed difference between cancer cases and matched controls [38,56]. Also, there was no difference in circulating selenium concentrations (nature log scale) between individuals with and without cancer (*p* value of *t* test = 0.16) in our study. Furthermore, we controlled for cancer status in our analyses to reduce the chance of false positive results. Some genomic regions may have gender-specific associations with serum selenium concentrations, in which case combined analysis of study participants in PLCO (all men) and in WHI (all women) may lead to false negative results. However, the vast majority of GWAS findings have been observed in men and women [57]. Given the differences between the study populations one may argue for separate analyses of both studies; however, this reduces the overall power and does not provide an opportunity to replicate findings. Therefore, we decided to conduct a combined analysis although it may be possible that more homogenous study populations would result in additional findings. This study focused on circulating selenium concentrations and did not examine the levels of the major plasma selenoproteins such as SEPP1 and GPX3. We identified two possible regions associated with serum selenium concentrations. If replicated, further work is needed to uncover the potential biological mechanisms underlying these associations. Our most significant SNPs with *p* < 5 × 10^−7^ did not reach the conventional genome-wide significant threshold (*p* < 5 × 10^−8^). Therefore, further studies are required to replicate our findings.

## 5. Conclusions

In conclusion, we performed a genome-wide association study on circulating selenium concentrations in two cohort studies and identified two potential regions, 4q34.3/*AGA*-*NEIL3* and 17q24.3/*SLC39A11*. Further investigations are needed to replicate the observed associations and reveal the biological mechanism by which SNPs influence selenium levels. In addition, larger genome-wide studies are needed to discover additional regions associated with circulating selenium concentrations.

## Supplementary Files

Supplementary File 1 Supplementary Information (PDF, 153 KB)
