# Supplementary material for: Genome-Wide Association Study of Serum Selenium Concentrations
Source: Nutrients. 2013 May 21;5(5):1706–18. doi: 10.3390/nu5051706 (PMC3708345; doi:10.3390/nu5051706)
Supplement: Supplementary File 1 — Supplementary Information (PDF, 153 KB) [file nutrients-05-01706-s001.pdf]

Supplementary Information

Figure S1. QuantilE−quantile plot (QQ plot) of the genomE−wide scan in PLCO (n = 582). The −log10 of p values for 2,474,333 SNPs were ranked in order from smallest to largest on the y-axis and plotted against the values expected under the null hypothesis of no SNP associated with serum selenium concentrations. The red line is the diagonal identity line. The inflation factor (λ) is 1.09.

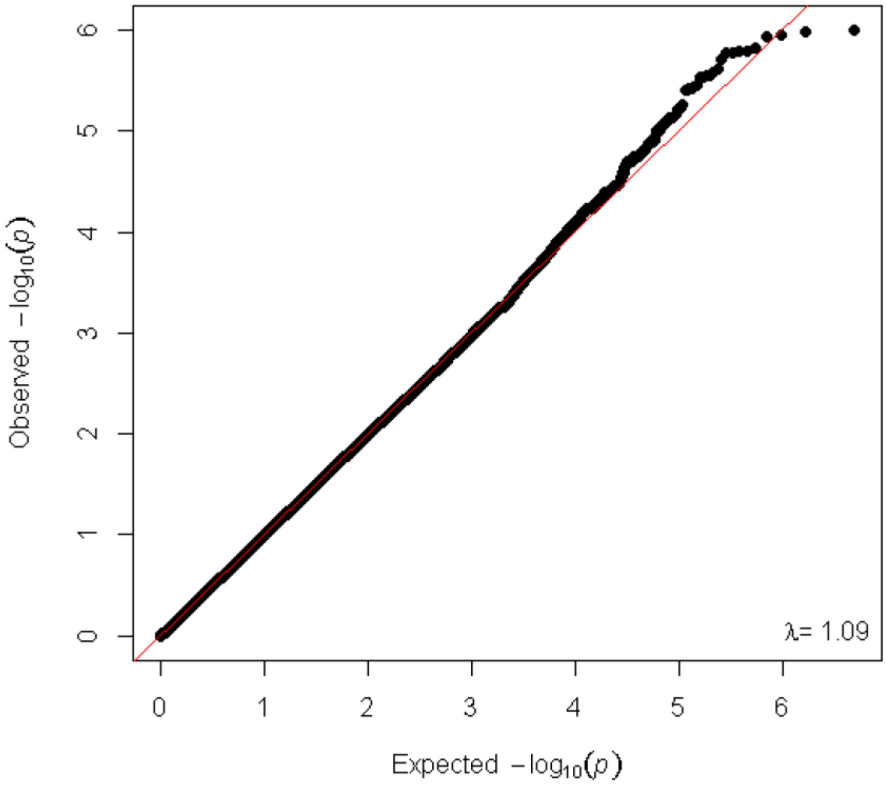

Table S1. The SNPs associated with serum selenium concentrations at $p < 1 \times 10^{-5}$ in the first & second stage analyses.

					PLCO only			WHI only			combined analysis			SNP_Imputed
**SNP_Name**	**Chr**	**BP (Build 36)**	**genes**	**region**	**beta**	**se**	***p* value**	**beta**	**se**	***p* value**	**beta**	**se**	***p* value**	
rs6701617	1	236735100	-	1q43	0.061	0.013	3.93E−06	0.018	0.010	8.34E−02	0.040	0.008	1.54E−06	1
rs6692700	1	236736399	-	1q43	0.061	0.013	3.82E−06	0.018	0.010	8.37E−02	0.040	0.008	1.51E−06	1
rs10925672	1	236736592	-	1q43	0.061	0.013	3.81E−06	0.018	0.010	8.29E−02	0.040	0.008	1.54E−06	1
rs6429079	1	236738917	-	1q43	−0.060	0.013	5.90E−06	−0.018	0.010	8.52E−02	−0.039	0.008	2.34E−06	1
rs12045395	1	236742025	-	1q43	−0.059	0.013	6.86E−06	−0.018	0.010	8.36E−02	−0.039	0.008	2.56E−06	1
rs10802701	1	236742138	-	1q43	0.059	0.013	7.00E−06	0.018	0.010	8.36E−02	0.039	0.008	2.61E−06	1
rs17016225	2	35224355	-	2p22.3	0.124	0.027	3.87E−06	−0.006	0.021	7.75E−01	0.060	0.017	4.19E−04	1
rs11902616	2	152175247	NEB	2q23.3	0.608	0.123	1.00E−06	−0.028	0.110	8.01E−01	0.344	0.083	3.43E−05	1
rs6717213	2	152184376	NEB	2q23.3	0.594	0.121	1.17E−06	−0.022	0.108	8.36E−01	0.340	0.081	3.23E−05	1
rs2372965	2	217675962	-	2q35	0.073	0.016	2.92E−06	0.008	0.011	4.88E−01	0.038	0.009	6.72E−05	1
rs2372967	2	217676158	-	2q35	0.073	0.016	2.90E−06	0.008	0.011	4.76E−01	0.037	0.009	6.72E−05	1
rs3843337	2	217677680	-	2q35	−0.073	0.015	2.58E−06	−0.008	0.011	4.72E−01	−0.038	0.009	6.04E−05	1
rs2372972	2	217679386	-	2q35	0.074	0.016	2.80E−06	0.009	0.011	4.09E−01	0.039	0.009	3.58E−05	0
rs9677455	2	217680497	-	2q35	−0.074	0.016	2.84E−06	−0.009	0.011	4.14E−01	−0.039	0.009	3.73E−05	1
rs1506807	*4*	*178554686*	-	*4q34.3*	*−0.059*	*0.013*	*8.39E−06*	*−0.025*	*0.010*	*1.33E−02*	*−0.043*	*0.008*	*2.63E−07*	*1*
rs1395479	*4*	*178555185*	-	*4q34.3*	*0.059*	*0.013*	*8.31E−06*	*0.025*	*0.010*	*1.33E−02*	*0.043*	*0.008*	*2.62E−07*	*0*
rs1445744	5	83493430	EDIL3	5q14.3	0.262	0.058	7.29E−06	0.023	0.041	5.76E−01	0.133	0.035	1.22E−04	1
rs17656910	5	154758983	-	5q33.2	0.129	0.028	5.43E−06	−0.005	0.021	8.25E−01	0.059	0.017	6.34E−04	1
rs17598647	5	154762275	-	5q33.2	−0.128	0.028	7.25E−06	0.005	0.020	8.20E−01	−0.057	0.017	7.98E−04	1
rs6891593	5	154985767	-	5q33.2	−0.131	0.029	7.47E−06	−0.003	0.020	9.00E−01	−0.058	0.017	6.30E−04	1
rs6884215	5	154990008	-	5q33.2	0.130	0.029	7.69E−06	0.003	0.020	8.97E−01	0.058	0.017	6.27E−04	1
rs6898274	5	155010159	-	5q33.2	−0.130	0.029	9.88E−06	−0.004	0.020	8.40E−01	−0.059	0.017	6.24E−04	1
rs17118166	5	155010282	-	5q33.2	0.130	0.029	9.83E−06	0.004	0.020	8.40E−01	0.059	0.017	6.21E−04	1
rs17118168	5	155012023	-	5q33.2	0.130	0.029	9.79E−06	0.004	0.020	8.40E−01	0.059	0.017	6.19E−04	1
rs9691085	7	82861302	SEMA3E	7q21.11	−0.121	0.025	1.51E−06	−0.001	0.018	9.49E−01	−0.054	0.015	4.51E−04	1
rs12532427	7	82867195	SEMA3E	7q21.11	0.107	0.024	9.31E−06	−0.002	0.017	9.02E−01	0.046	0.015	1.66E−03	1
rs12536989	7	82909807	SEMA3E	7q21.11	0.117	0.024	1.12E−06	0.000	0.018	9.84E−01	0.052	0.015	4.16E−04	0

Table S1. *Cont.*

rs4875284	8	3965538	CSMD1	8p23.2	−0.347	0.072	1.96E−06	−0.080	0.050	1.12E−01	−0.201	0.043	3.13E−06	1
rs4841058	8	8768326	MFHAS1	8p23.2	0.891	0.187	2.46E−06	0.038	0.093	6.85E−01	0.292	0.090	1.23E−03	1
rs1545157	10	9298702	-	10p14	−0.058	0.013	8.53E−06	0.004	0.010	6.84E−01	−0.027	0.008	1.12E−03	0
rs10905530	10	9308128	-	10p14	0.063	0.013	3.56E−06	0.000	0.010	9.66E−01	0.031	0.008	2.50E−04	1
rs1149958	10	9319090	-	10p14	0.066	0.014	3.70E−06	0.001	0.011	9.53E−01	0.033	0.009	1.98E−04	1
rs4765709	12	2753898	-	12p13.33	−0.061	0.013	1.59E−06	0.005	0.009	5.89E−01	−0.027	0.008	4.93E−04	1
rs4073984	12	2756041	-	12p13.33	0.062	0.012	1.04E−06	−0.004	0.009	6.78E−01	0.027	0.008	3.09E−04	0
rs4964010	12	26689656	ITPR2	12p11.23	0.056	0.013	8.34E−06	0.011	0.010	2.53E−01	0.034	0.008	1.56E−05	1
rs11655160	17	10833795	-	17p12	−0.078	0.017	6.18E−06	−0.001	0.012	9.54E−01	−0.038	0.010	3.16E−04	1
rs11650930	17	10833819	-	17p12	0.078	0.017	6.14E−06	0.000	0.012	9.71E−01	0.038	0.010	3.38E−04	1
rs891684	*17*	*68225134*	*SLC39A11*	*17q24.3*	*−0.133*	*0.030*	*8.73E−06*	*−0.057*	*0.022*	*9.98E−03*	*−0.093*	*0.018*	*4.04E−07*	*1*
rs6000096	22	34795642	-	22q12.3	18.819	3.891	1.70E−06	0.124	0.167	4.56E−01	0.190	0.193	3.25E−01	1
rs5995206	22	34796910	-	22q12.3	18.819	3.891	1.70E−06	0.124	0.167	4.56E−01	0.190	0.193	3.25E−01	1
rs6000109	22	34802098	-	22q12.3	17.825	3.680	1.64E−06	0.128	0.167	4.45E−01	0.197	0.194	3.08E−01	1

Note: “-” indicates intergenic; SNP imputed: 1 imputed, 0 genotyped.
